# Formulation and In Vitro and In Silico Characterization of “Nano-in-Micro” Dry Powder Inhalers Containing Meloxicam

**DOI:** 10.3390/pharmaceutics13020211

**Published:** 2021-02-03

**Authors:** Petra Party, Csilla Bartos, Árpád Farkas, Piroska Szabó-Révész, Rita Ambrus

**Affiliations:** 1 Interdisciplinary Excellence Centre, Institute of Pharmaceutical Technology and Regulatory Affairs, University of Szeged, Eötvös street 6, 6720 Szeged, Hungary; party.petra@szte.hu (P.P.); bartos.csilla@szte.hu (C.B.); revesz@pharm.u-szeged.hu (P.S.-R.); 2 Centre for Energy Research, Hungarian Academy of Sciences, Konkoly-Thege Miklós Street 29-33, 1121 Budapest, Hungary; farkas.arpad@energia.mta.hu

**Keywords:** dry powder inhaler, nano, meloxicam, wet milling, spray-drying, Andersen cascade impactor, in silico assessment

## Abstract

Pulmonary delivery has high bioavailability, a large surface area for absorption, and limited drug degradation. Particle engineering is important to develop inhalable formulations to improve the therapeutic effect. In our work, the poorly water-soluble meloxicam (MX) was used as an active ingredient, which could be useful for the treatment of non-small cell lung cancer, cystic fibrosis, and chronic obstructive pulmonary disease. We aimed to produce inhalable “nano-in-micro” dry powder inhalers (DPIs) containing MX and additives (poly-vinyl-alcohol, leucine). We targeted the respiratory zone with the microcomposites and reached a higher drug concentration with the nanonized active ingredient. We did the following investigations: particle size analysis, morphology, density, interparticular interactions, crystallinity, in vitro dissolution, in vitro permeability, in vitro aerodynamics (Andersen cascade impactor), and in silico aerodynamics (stochastic lung model). We worked out a preparation method by combining wet milling and spray-drying. We produced spherical, 3–4 µm sized particles built up by MX nanoparticles. The increased surface area and amorphization improved the dissolution and diffusion of the MX. The formulations showed appropriate aerodynamical properties: 1.5–2.4 µm MMAD and 72–76% fine particle fraction (FPF) values. The in silico measurements proved the deposition in the deeper airways. The samples were suitable for the treatment of local lung diseases.

## 1. Introduction

The main advantages of pulmonary delivery are the result of the huge surface area of the lung (100 m^2^) with a thin absorption layer (0.1–0.2 µm), as well as low metabolic activity. Targeted delivery of the drug could provide benefits such as achieving a greater local concentration at the target site with a reduced dose, resulting in reduced systemic side effects and adverse events [1]. Local delivery is especially effective in patients with serious pulmonary diseases such as asthma, cystic fibrosis (CF), chronic obstructive pulmonary (COPD) disease, and lung cancer [2].

For the application of inhaled medications, dry powder inhalers (DPIs) are more widely used compared with nebulizers or metered-dosed inhalers (MDIs). DPI products are solid-state; therefore, they have long-term stability. The delivery is driven by the inhalation flow, thus DPIs are environmentally friendly, and they do not require a compressor or propellant. The administration time is very short and the devices are cheap and portable [3]. Unfortunately, drug deposition in the pulmonary region is not sufficient with traditional carrier-based DPIs. In these systems, the active ingredient is attached to the surface of a carrier, which is usually lactose, although it could be mannitol or glucose too. The potentiality of the powders is proper dispersion in the respiratory system, so the aerosolization of the products should be optimized. Hence, new carrier-free DPI systems have been developed to enhance the therapeutic effect. To reach efficient deposition, DPIs should contain a powder made of the active pharmaceutical ingredient (API) co-formulated with appropriate excipients, which are chosen based on their functions in the powder, leading to optimal aerodynamic properties [4]. Excipients approved for DPI formulations are, for example, hydrophobic additives (Mg-stearate) for protection against moisture, lipids (cholesterol) for coating, amino acids (leucine) for improved aerosol efficiency, and absorption enhancers (cyclodextrins, chitosan) and biodegradable polymers (poly(lactic-co-glycolic acid) (PLGA) for stability and released formulations [2].

Besides the components, the particle size and dispersibility of DPIs have a key role in the deposition pattern. There are three principal mechanisms of particle deposition in the lung. Inertial impaction affects particles that are larger than 5 µm. These particles are not able to follow the changes of gas flow direction in the upper airway and at the airway bifurcations. Therefore, the particles impact the upper airways walls, limiting the amount of API that can be delivered into the lung. Gravitational sedimentation is based on the settling of particles under the action of gravity and occurs in the smaller airways and where the distance is covered by the particles before they hit the wall of the airways. This deposition mechanism is the most effective for particles in the size range of 1–8 µm. DPIs in this size range are best suited to treat central and small airways. Random motions of the particles caused by their collisions with gas molecules result in deposition by Brownian diffusion. Unlike deposition by impaction and sedimentation, which increase with the increasing particle size, deposition by Brownian diffusion rises with decreasing particle size and becomes the dominant mechanism of deposition for particles less than 1 µm in diameter. These particles are effective in the alveolar region of the lung, where air velocities are low [5]. Particles under 1 µm usually are exhaled. In conclusion, the requested particle size range in pulmonary therapy is a particle diameter of 1–5 µm.

Nanoparticles are a beneficial formulation for Class II drugs of the Biopharmaceutics Classification System (BCS), where the dissolution rate is the rate-limiting step for absorption. The reduction of particle size can increase the dissolution rate as the amount of API dissolving over time is inversely correlated with the particle diameter. For this reason, nanoparticle formulations of API are being assessed for their potential to increase the drug dissolution rate as a result of a higher specific surface area. If we formulate the nanosized API into micrometric particles, we can target the proper parts of the airways and, when the powders come into contact with the lung lining fluid, the particles can disintegrate into their nano subunits and spread on the surface of the epithelium, resulting in a large surface area for drug dissolution, and thus increased absorption and more homogenous distribution [6]. A prosperous formulation for nanoparticle agglomerates is the preparation of nanosuspensions by wet milling followed by solidification, using spray-drying. They are reproducible, scalable, and cost- and time-effective preparation methods. We can combine the advantages of nanonized particles by preparing a nanosuspension (i.e., enhanced dissolution and solubility) with the benefits of solid formulations (i.e., stability, easier handling, and enhanced patient compliance) by producing microsized nanoparticle agglomerates suitable for pulmonary delivery [7].

Our research group had experiences with meloxicam (MX) as an API and different additives, such as polymers and amino acids. In this work, we used MX, which is a poorly water-soluble (in water, 7.15 mg/L at 25 °C), non-steroidal anti-inflammatory agent [8]. In pulmonary therapy, it could be useful to treat CF, COPD, and non-small-cell lung cancer [9,10,11,12]. Previous studies were about particle size reduction of meloxicam with wet milling using poly-vinyl-alcohol (PVA) solution as a dispersant [13]. In the presence of PVA, the particle size of the drug could be reduced to the nanometre range. In the case of co-spray-dried DPI formulations, PVA exerted an aggregation inhibitor effect, thereby providing individual particles [14]. l-leucine (LEU) was applied to enhance the dispersity of the particles, thereby improving the aerosolization and the flowability of the powders [15,16]. Our works correlated with the positive effect of LEU on the aerodynamic properties, because LEU decreases the deposition in the upper airways and increases the emitted fraction during inhalation [17,18].

In the following work, we formulated micrometer-sized carrier-free DPIs using spray-drying, containing the previously nanonized active ingredient by wet milling. The novelty of the present work is the “nano-in-micro” structure of the DPI. We carried out morphology, rheology, structure, dissolution, diffusion, and aerodynamic characterization of the samples. We wanted to target the respiratory zone with micrometric particles. Thanks to the particle size reduction of the poorly water-soluble MX, and thus the increase of the specific surface area, we could improve the local dissolution in the lung fluid and permeability to the epithelium. Our product could provide an effective treatment for serious local pulmonary diseases.

## 2. Materials and Methods

### 2.1. Materials

Meloxicam (MX) (Egis Pharmaceuticals PLC., Budapest, Hungary) was used as an active ingredient. As additives, poly-vinyl-alcohol 3-88 (PVA) (ISP Customer Service GmBH, Cologne, Germany) and l-leucine (LEU) (AppliChem GmbH, Darmstadt, Germany) were applied.

### 2.2. Preparation Method

**Figure 1:**
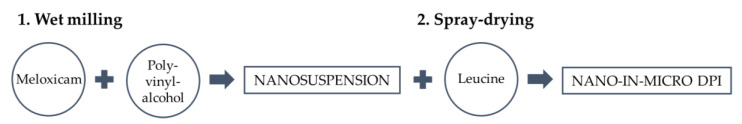
Two-step preparation method of the samples. DPI, dry powder inhaler.

We used a two-step preparation protocol. First, the pre-nanosuspension was prepared by wet milling technology, using PVA and MX. The final microsized powders were obtained with co-spray drying of the diluted suspension and LEU (Figure 1).

#### 2.2.1. Wet Milling

We applied a combined wet milling technique, which was optimized by our research group’s previous work [13]. We dissolved 2.5 g of PVA in purified water and the volume of the final solution was 100 mL. Then, 2.00 g of MX was suspended in 18.0 g of 2.5% (mass/volume) PVA solution. Moreover, 20.0 g of ZrO_2_ beads was the milling medium in a planetary ball mill (Retsch Planetary Ball Mill PM 100 MA, Retsch GmbH, Haan, Germany). The milling parameters were as follows: 60 min and 500 rpm. As the result of the wet milling, we achieved a nanosized pre-suspension containing MX and PVA. The nanosuspension was diluted with purified water to 500 mL. The final concentration of the MX suspension was 4 g/L.

#### 2.2.2. Co-Spray Drying

We prepared different compositions by adding a various amount of LEU, as shown in Table 1. A magnetic stirrer was used for sample homogenization (AREC.X heating magnetic stirrer, Velp Scientifica Srl, Usmate Velate, Italy). The inhalable microparticles were produced by spray-drying using a spray-dryer equipped with a two-fluid nozzle of 0.7 mm (Büchi Mini Spray Dryer B-191, Büchi, Flawil, Switzerland). Based on the preliminary experiments, the spray drying properties were as follows: inlet temperature: 165 °C, outlet temperature: 100 °C, aspirator capacity: 85%, airflow rate: 500 L/h, and feed pump rate: 10%. The yield was calculated as the ratio of the mass of the particles collected after spray-drying to the mass of the solid content of the initial nanosuspension. We managed to increase the yield of spray-drying with the addition of LEU. Low spray-drying yields are indicative of cohesive powders. LEU reduced the cohesion between the particle, hence the improvement of the spray-drying yield [19].

#### 2.2.3. Physical Mixtures

We prepared physical mixtures of the raw materials. The compositions were the same as for the spray-dried samples (Table 2). During our measurements, we compared the properties of the spray-dried samples to the those of the physical mixtures.

### 2.3. Determination of Particle Size and Distribution

Laser diffraction was used to determine the particle size and the particle size distribution of our samples (Malvern Mastersizer Scirocco 2000, Malvern Instruments Ltd., Worcestershire, UK). The wet dispersion unit was used to measure the particle size of the nanosuspension. We set the refractive index of MX (1.720) and measured it in purified water with 2000 rpm stirring. The dry dispersion unit was used to observe the spray-dried microcomposites. Approximately 0.5–1.0 g of product was loaded into the feeding tray. The dispersion air pressure was adjusted to 3.0 bar and 75% vibration feed was used. Each sample was measured in triplicate. The particle size distribution was characterized by the D[0.1] (10% of the volume distribution is below this value), D[0.5] (the volume median diameter is the diameter where 50% of the distribution is above and 50% is below), and D[0.9] (90% of the volume distribution is below this value) values. The size distribution Span was calculated according to Equation (1). A high Span value denotes a broad particle size distribution. The higher the Span value, the broader the particle size distribution [20]. We obtained the specific surface area (SSA) data, which predict the dissolution and permeability properties of the samples.

(1) $$\mathrm{Span}=\frac{D[0.9]-D[0.1]}{D[0.5]}$$

### 2.4. Investigation of Morphology

Scanning electron microscopy (SEM) (Hitachi S4700, Hitachi Scientific Ltd., Tokyo, Japan) was used to characterize the morphology of the spray-dried formulation. We applied a high voltage of 10 kV, an amperage of 10 mA, and an air pressure of 1.3–13.1 mPa. A high vacuum evaporator and argon atmosphere were used to make the sputter-coated samples conductive with gold-palladium (Bio-Rad SC 502, VG Microtech, Uckfield, UK). The thickness of the gold-palladium coating was approximately 10 nm. For the particle size analysis of the active ingredient, a public domain image analyzer software, ImageJ, was used (https://imagej.nih.gov/ij/index.html).

### 2.5. Density Measurement

The bulk and tapped densities of the formulations were measured using an Engelsmann Stampfvolumeter (Ludwigshafen, Germany) [21]. A 10 cm^3^ cylinder was filled with 1.5–2.0 cm^3^ of powder to calculate bulk density. Then, it was tapped 1000 times. The tapped density of the samples was calculated compared with the volume before and after the taps. We calculated the flow characters (Equations (2) and (3)) of the samples from the bulk (ρ_b_) and tapped (ρ_t_) density. All samples were measured in triplicate.

(2) $$\mathrm{Hausner}\;\mathrm{ratio}=\frac{\rho_{t}}{\rho_{b}}$$

(3) $$\mathrm{Carr}\;\mathrm{index}=\frac{\rho_{t}-\rho_{b}}{\rho_{t}}*100$$

### 2.6. Determination of the Interparticle Interactions

Around 0.10 g of the samples was pressed on a 1 ton hydraulic press (Perkin Elmer hydraulic press, Specac Inc., Waltham, MA, USA). Six pastilles were obtained from each sample. We did three parallel measurements with each composition. Three pastilles per sample were dripped with polar liquid (4.8 µL of purified water) and the other three pastilles were dripped with non-polar solvent (2.0 µL of diiodomethane). Contact angle was detected in an interval of 1 to 25 s with a Dataphysics OCA 20 apparatus (Dataphysics Instrument GmbH, Filderstadt, Germany) [22]. We obtained the contact angles of the two applied fluids. The surface free energy (γ_s_) of the composites, which consists of the polar part (γ_s_^p^) and the disperse part (γ_s_^d^), so (γ_s_ = γ_s_^p^ + γ_s_^d^), was calculated based on the Wu equation. The surface tension of the used liquids is known in the literature: distilled water γ^p^ = 50.2 mN/m, γ^d^ = 22.6 mN/m and diiodomethane γ^p^ = 1.8 mN/m, γ^d^ = 49 mN/m. We can express the Wu equation (Equation (4)), where θ = contact angle, γ = surface free energy, s = solid phase, l = liquid phase, d = dispersion component, and p = polar component.

(4) $$\left(1+\cos\theta \right)\gamma_{l}=\frac{4{\gamma_{s}}^{d}{\gamma_{l}}^{d}}{{\gamma_{s}}^{d}+{\gamma_{l}}^{d}}+\frac{4{\gamma_{s}}^{p}{\gamma_{l}}^{p}}{{\gamma_{s}}^{p}+{\gamma_{l}}^{p}}$$

Polarity (Pol) was calculated as the ratio of the surface free energy of the polar component and surface free energy multiplied by 100 (Equation (5)).

(5) $$\mathrm{Pol}=\frac{\gamma^{p}}{\gamma^{s}}*100$$

Cohesion work (W_c_) was determined as twice the surface free energy (Equation (6)).

(6) $$W_{c}=2*\gamma_{s}$$

### 2.7. Structural Analysis

To establish the crystalline character of the spray-dried samples, X-ray powder diffraction (XRPD) spectra were recorded with a BRUKER D8 Advance X-ray diffractometer (Bruker AXS GmbH, Karlsruhe, Germany). The radiation source was Cu Kλ1 radiation (λ = 1.5406 Å). Measurement conditions were as follows: Cu target, Ni filter, 40 kV voltage, 40 mA current, time constant 0.1°/min, and angular step 0.010° over the interval 3–40°. We used the DIFFRACT plus EVA 28 software (Bruker AXS GmbH, Karlsruhe, Germany) for the evaluation.

### 2.8. Thermoanalitycal Analysis

The differential scanning calorimetry (DSC) measurements were made with a Mettler Toledo DSC 821^e^ thermal analysis system with the STAR^e^ thermal analysis program V9.1 (Mettler Inc., Schwerzenbach, Switzerland). Approximately 2–5 mg of the samples was examined in the temperature range between 25 °C and 300 °C. The heating rate was 5 °C/min. Argon was the carrier gas at a flow rate of 10 L/h during the investigation.

### 2.9. In Vitro Dissolution Test

No in vitro dissolution test for powders for inhalation exists in the current Pharmacopeia. We applied a modified paddle method (Hanson SR8 Plus, Teledyne Hanson Research, Chatsworth, CA, USA) from the European Pharmacopeia [23] to examine the release of MX from the samples. The capacity of the vessel was 100 mL instead of 1000 mL and the size of the stirrer was smaller. We designed the parameters of our measurement based on the circumstances of the human airways [24]. The medium was a simulated lung medium, which contained NaCl, NaHCO_3_, CaCl_2_, NaH_2_PO_4_, H_2_SO_4_, and glycine [25]. The volume of the medium was 50 mL based on the estimated volume of the lung fluid [26]. The pH of the medium was 7.4 ± 0.1. The temperature was set at 37 °C. The samples contained 1.5 mg of MX, which is one-tenth of the oral dose of the API. During pulmonary delivery, we can reduce the amount of API compared with the oral dose. We chose this amount of API based on a salbutamol dosage recommendation [27]. Applying these doses of our products is safe for use. Previous investigations proved that the API and the excipients had no cytotoxic effect on the concentration on the cells [28]. The paddle was rotated at 100 rpm and the sampling was performed up to 60 min. The total fraction of the samples was dispersed in the medium. We took 5 mL of the dissolution medium after 5, 10, 15, 30, and 60 min. The medium was replenished every time the sample was withdrawn. After filtration (pore size: 0.45 µm, Millex-HV syringe-driven filter unit, Millipore Corporation, Bedford, MA, USA) and dilution, the MX contents of the samples were determined by spectrophotometry at λ = 362 nm (ATI-UNICAM UV/VIS Spectrophotometer, Cambridge, UK). Three parallel measurements took place with the formulations.

### 2.10. In Vitro Diffusion Test

We would like to demonstrate the permeability from the lung fluid to the epithelial cells of the lung. A modified horizontal diffusion cell was used to investigate the in vitro permeability of the samples [26]. The donor phase (9 mL) was simulated lung medium (pH = 7.4). Phosphate buffer (pH = 7.4) was used as the acceptor phase (9 mL), modelling the circumstances of the epithelial cell. Between the two phases, there was a cellulose membrane (RC 55 Whatman^TM^ GE Healthcare Life Sciences, Buckinghamshire, UK) impregnated with isopropyl myristate. The actual diffusion surface was 0.785 cm^2^. The rotation of the stirring bar was set to 300 rpm. The temperature was 37 °C. We measured 1.5 mg MX contents of the samples. The API was first released in the simulated lung fluid, and then diffused through the membrane to the phosphate buffer. The amount of diffused MX was determined real-time at λ = 362 nm until 60 min with sonda (FDP-7UV200-VAR, Avantes, Apeldoorn, The Netherlands) spectrophotometer (Avaspec-ULS2048-USB2, Avantes, Apeldoorn, The Netherlands) in the acceptor phase [29]. The samples were measured three times.

The flux (J) [µg/cm^2^/h] of the active ingredient was calculated from the quantity of MX, which permeated through the membrane, divided by the surface of the membrane insert (A) and the duration (t) using the following Equation (7):

(7) $$J=\frac{m}{A*t}$$

The permeability coefficient (Kp) [cm/h] was determined from the flux and the MX concentration in the donor phase (C_d_) [µg/cm^3^], Equation (8):

(8) $$K_{p}=\frac{J}{C_{d}}$$

### 2.11. In Vitro Aerodynamic Measurements

The aerosolization efficacy of the spray-dried formulations was assessed in vitro, using an Andersen cascade impactor (ACI) (Apparatus D, Copley Scientific Ltd., Nottingham UK) [30]. The inhalation flow rate was set to 28.3 ± 1 L/min (High-capacity Pump Model HCP5, Critical Flow Controller Model TPK, Copley Scientific Ltd., Nottingham, UK). Table 3 shows the cut-off aerodynamic diameter for stages of ACI at a flow rate of 28.3 L/min [31]. The actual flow rate through the impactor was measured by a mass flow meter (Flow Meter Model DFM 2000, Copley Scientific Ltd., Nottingham, UK). The inhalation time was 4 s for one inhalation. These parameters led to an inhalation volume of 1.89 L, which was similar to the inhalation volume of COPD patients [32]. Breezhaler^®^ single dose devices (Novartis International AG, Basel, Switzerland) were used, with transparent size 3 gelatine capsules (Capsugel, Bornem, Belgium) filled with 2.0–2.5 mg of powder, which contained 1–2 mg of MX. Four capsules were inhaled during one measurement. The inhaler was actuated twice for each capsule. Each sample was measured in triplicate.

To provide the pulmonary adhesive circumstances, the plates on the stages were coated with Span 85 and cyclohexane (1 + 99 *w*/*w*%) mixture. After inhalation, the device, the capsules, the induction port, the collection plates, and the filter were washed with methanol and pH 7.4 phosphate buffer (60 + 40 *v*/*v*%) to collect the deposited MX. The collected and dissolved MX was quantified by UV/vis spectrophotometry (ATI-UNICAM UV/VIS Spectrophotometer, Cambridge, UK) at a wavelength of λ = 362 nm.

The actual API content (%) of the spray-dried particles was measured by dissolving 1.0–1.1 mg of product in 25 mL of methanol/phosphate buffer (60:40 *w*/*w*%), the solution of which was also used for the aerodynamic measurement. The solutions were mixed for 10 min at 600 rpm, and the API content was quantified by UV/vis spectrophotometry (ATI-Unicam UV/VIS Spectrophotometer, Cambridge UK) at a wavelength of 362 nm.

The aerodynamic properties were calculated from a plot of the cumulative percentage undersize of the API on log probability scale against the effective cut-off diameter using the KaleidaGraph program [31,33]. The mass of drug particles with a size under 5 µm was defined as a fine particle dose (FPD). The amount of drug leaving the device and reaching the impactor was considered as the emitted dose (ED). The fine particle fraction (FPF) was calculated as the percentage ratio between FPD and ED. The emitted fraction (EF) was expressed as a percentage of the ED divided by the initial amount of API. The aerodynamic diameter is influenced by the inhalation flow rate, density, and size and shape of the particle. The real size of the particle during inhalation is expressed with the MMAD (median mass aerodynamic diameter). The MMAD of the particles was determined from the same plot as the particle size corresponding to the 50% point of the cumulative distribution. For an inhalable and well-deposited powder, the MMAD should be in the 1–5 µm size range [34].

### 2.12. In Silico Characterization

The in silico simulations were performed by the stochastic lung deposition model, which tracks the inhaled particles until their deposition or exhalation and computes the fraction of the particles deposited in each anatomical part of the respiratory system, that is, extrathoracic, bronchial, and acinar regions [35]. The particle trajectories were simulated in an asymmetrical branching airway structure, mimicking the realistic airways by selecting morphometrical parameters from the database of Raabe et al. [36]. The inputs of the computational model are different parameters characterizing the aerosol particles like density, shape, or size, as well as the breathing parameters of the patient, such as inhaled volume, inhalation time, breath-hold time, exhalation time, and breathing mode (nasal or oral). A more detailed description of the numerical model can be found in Koblinger and Hofmann [37]. In our work, aerodynamic particle size distributions of the samples measured by the Andersen cascade impactor technique served as the inputs for the numerical airway deposition model. The inhalation parameters corresponded to a COPD patient inhaling through Breezhaler^®^, whose inhaled volume (IV) and inhalation time values (IV = 1.7 L, t_in_ = 3.2 s) matched the best flow rate of the current impactor measurements. The computational deposition model was validated for the case of aerosol drugs in our earlier works [38,39].

## 3. Results

### 3.1. Particle Size Distribution

We managed to prepare a nanosuspension using raw MX and 2.5% PVA dilution during the milling procedure. In the diluted suspension, the particle size of MX was 137.70 nm ± 4.965 nm and the SSA was 43.65 ± 5.318 m^2^/g. After spray-drying, the size of the particles was applicable for pulmonary delivery, as the D [0.5] values were in the 1–5 µm range and the distribution was monodisperse (Table 4). The geometric diameter of spray-dried nanoMX1_LEU0 was around 3.2 μm. Incorporating LEU in the formulations increased the geometric size of the spray-dried particles [40]. The distribution was monodisperse in all cases (Span < 2.0), which is essential for accurate dosing. The specific surface area (SSA) values increased compared with the raw materials, which predicted an improved dissolution profile.

### 3.2. Particle Morphology

The SEM pictures (Table 5) showed particles with a nearly spheroidal shape, which was the result of the optimized parameters of the co-spray-drying. The particles were produced from the droplets during the method, and the most stable shape for a droplet is the spherical form [41]. According to the SEM pictures, we observed that the presence of PVA prevented the aggregation. Particles were individually separated and displayed a regular size, which met the requirements for the formulation of a DPI [14]. The peclet number of LEU is greater than 1, which led to a wrinkled particle morphology after spray-drying. This rough surface improved the dispersion of the particles, reflecting low density and resulting in higher drug delivery into the low regions of the airways [18,40,42]. We could observe the nanosized active ingredient particles in the SEM pictures. We measured the diameter of the API with Image-J program. The size range of these was between 120 and 140 nm (Table 4). The diameter of the MX was correlated with the results of the nanosuspension. The images proved the “nano-in-micro” structure of the final powders.

### 3.3. Powder Rheology

The lower density of DPI particles could offer better flowability and improved deposition within the deeper airways. Thanks to the additives, the density was reduced to under 0.3 g/cm^3^. The usual density of DPIs is about 1 g/cm^3^, so our samples can be considered as low-density formulation. The lower the tap density (0.04–0.25 g/cm^3^), the greater the respirable fraction [43]. The higher amount of LEU included in the sample nanoMX1_LEU1 was found to further reduce density, as demonstrated by the more wrinkled appearance of the particles [17]. The Hausner ratio (HR) was between 1.4 and 1.8. The Carr index (CI) results were in the range of 27 and 43 (Table 6). The result indicates poor flowability, but it is similar to other carrier-free formulations in the literature [44]. HR and CI values are also responsible for the aerosolization performance [45].

### 3.4. Interparticular Interactions

Contact angle measurements were performed to calculate the polarity and the cohesive work (Wc) characteristic of the materials. The wettability study revealed that the microcomposites had a more hydrophilic character as compared with hydrophobic MX. With the use of PVA, the polarity increased, which predicted better dissolution results in simulated lung medium compared with raw MX. The highest polarity values were obtained with nanoMX1_LEU0. The lipophilic component LEU decreased the polarity of the samples. In the case of samples containing LEU, cohesivity decreased between the spherical, rough particles, so the presence of LEU caused the decrease in W_c_ (Table 7). The lower cohesivity of particles could result in more effective deposition properties.

### 3.5. X-ray Powder Diffraction Results

**Figure 2:**
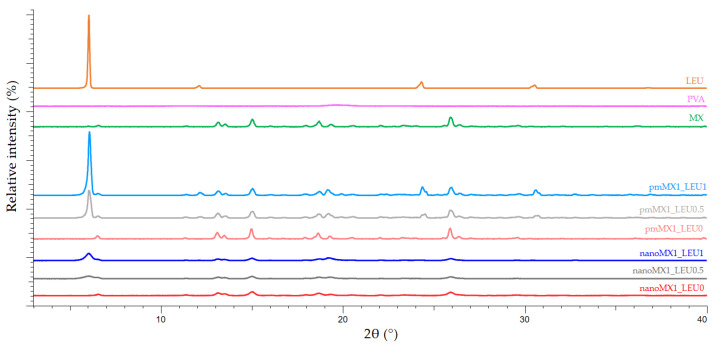
X-ray powder diffraction (XRPD) results of the raw materials (l-leucine (LEU), poly-vinyl-alcohol (PVA), and meloxicam (MX)), the physical mixtures (pmMX1_LEU0, pmMX1_LEU0.5, and pmMX1_LEU1), and the spray-dried samples (nanoMX1_LEU0, nanoMX1_LEU0.5, and nanoMX1_LEU1).

X-ray powder diffraction was used to characterize the crystalline state of MX after the preparation process. The XRPD pattern of the raw materials demonstrated the crystalline structure of MX and LEU, as expected. Raw MX has characteristic peaks with the highest intensities at 6.6°, 11.4°, 13.1°, 13.5°, 15.1°, 18.7°, 19.3°, 25.9°, and 26.4° 2-theta peaks, indicating its crystalline structure [46]. We detected the characteristic peaks of LEU at 6.12, 24.39, and 30.61 2-theta peaks [47]. In the case of the products, the intensities of the characteristic peaks decreased (Figure 2). The presence of PVA had no effect on the diffractograms. In the course of milling and spray-drying, a decrease in crystallinity was perceptible, which was determined via the mean of the decrease of the total area beneath the curve of the characteristic peaks compared with the physical mixtures. After treatment, ~71% of MX remained crystalline for nanoMX1_LEU0, ~52% for nanoMX1_LEU0.5, and ~53% for nanoMX1_LEU1. The other part of the active ingredient became amorphous during the preparation process. The preliminary stability test showed no changes in the structure after one month.

### 3.6. Thermoanalytical Results

**Figure 3:**
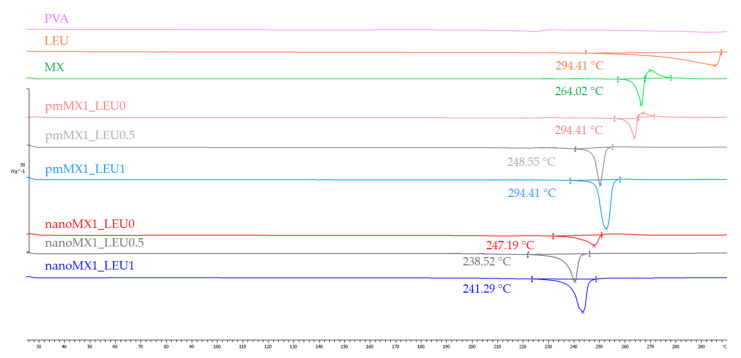
DSC results of the raw materials, (PVA, LEU, and MX), the physical mixtures (pmMX1_LEU0, pmMX1_LEU0.5, and pmMX1_LEU1), and the spray-dried samples (nanoMX1_LEU0, nanoMX1_LEU0.5, and nanoMX1_LEU1).

DSC was employed to investigate the melting of PVA, LEU, and MX in the raw form, in the physical mixtures, and in the prepared products (Figure 3). PVA had no endothermic peak. LEU had an endothermic peak at 294.41 °C. The DSC curves of raw MX showed a sharp endothermic peak at 264.03 °C, reflecting its melting point and crystalline structure. After milling and spray drying, the DSC curves in all cases exhibited broader endothermic peaks of MX, indicating that the crystallinity of the drug decreased. The residual MX crystals in the products melted at a lower temperature than the crystals of raw MX owing to the smaller particle size and the increased degree of amorphization. This was promoted by PVA, which was softened at 85 °C as the glass transition temperature (T_g_) value.

### 3.7. In Vitro Dissolution Results

**Figure 4:**
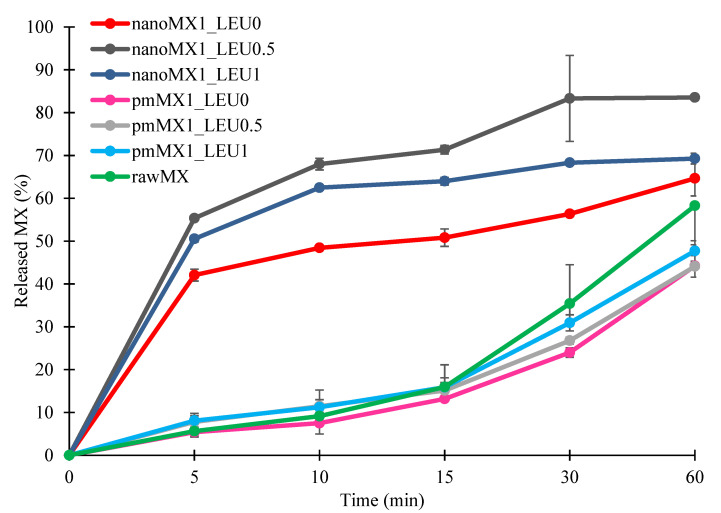
In vitro dissolution results of the active pharmaceutical ingredient (API) (rawMX), the physical mixtures (pmMX1_LEU0, pmMX1_LEU0.5, and pmMX1_LEU1), and the prepared samples (nanoMX1_LEU0, nanoMX1_LEU0.5, and nanoMX1_LEU1). Data are means ± SD (n = 3 independent measurements).

The initial API showed poor water-solubility, as we mentioned above. The formulations were compared to raw MX and the physical mixtures. The results of the dissolution study confirmed our predictions (Figure 4). The released amount of MX was the lowest in the case of samples containing raw material during the investigation. The spray-dried samples showed enhanced drug release compared with the reference samples. Approximately half of the drug was released from the samples containing nanonized API within the first 5 min and 65–85% released within an hour. These improvements in dissolution profile could be related to nanosizing effects, higher specific surface area, and amorphization. The presence of PVA inhibited the aggregation and increased polarity, which helped to release the MX in the simulated lung medium. Applying LEU reduced the cohesion between the particles, so a larger amount of MX was liberated from the powder than without LEU. The highest amount of API was released from nanoMX1_LEU0.5, because the higher LEU concentration reduced the polarity of the products. The results of our formulations are promising in the local pulmonary therapy. The prolonged presence of the particles gives enough time to release the nanosized API. Therefore, the clearance mechanism of the lung will reduce the delivered drug dose by a lesser amount [6].

### 3.8. In Vitro Permeability Results

**Figure 5:**
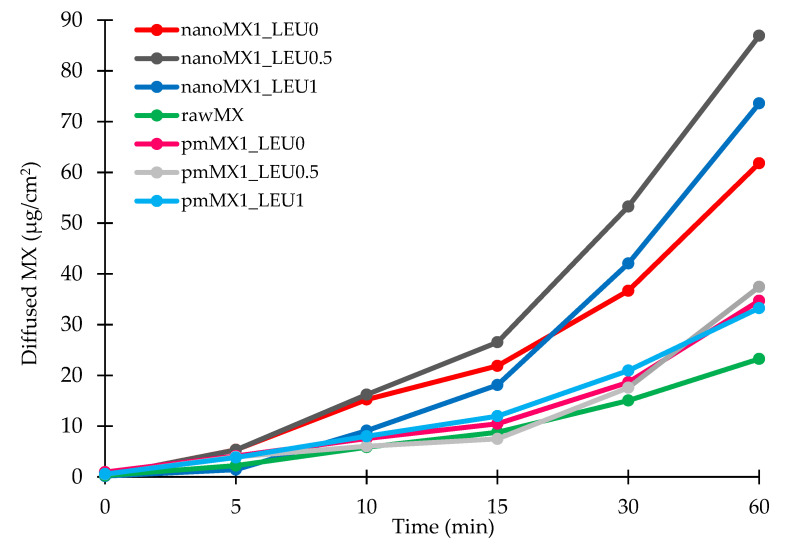
In vitro diffusion results of the API (rawMX), the physical mixtures (pmMX1_LEU0, pmMX1_LEU0.5, and pmMX1_LEU1), and the prepared samples (nanoMX1_LEU0, nanoMX1_LEU0.5, and nanoMX1_LEU1). SD < ±2% (n = 3 independent measurements).

We investigated the diffused amount of API from the simulated lung medium through a membrane to the epithelium. The high surface area achieved by the nanosized particles was the main factor affecting the rate of passive diffusion. Diffusion from the samples was faster and reached higher values than from raw MX and the physical mixtures (Figure 5). The diffused MX concentrations (60–90 µg/cm^2^) were promising if we interpolate them to the total surface of the lung. We reached the highest values with the nanoMX1_LEU0.5 formulation, which was correlated with the result of the in vitro dissolution test. The products showed a significantly increased flux (J) and permeability coefficient (Kp) compared with the raw materials (Table 8). The higher diffusion is connected to the higher surface area produced by the nanoparticles. A large amount of API could get into the epithelium with our spray-dried formulations; as a result of this, they could be effective in the local treatment of pulmonary diseases.

### 3.9. In Vitro Aerodynamic Results

**Figure 6:**
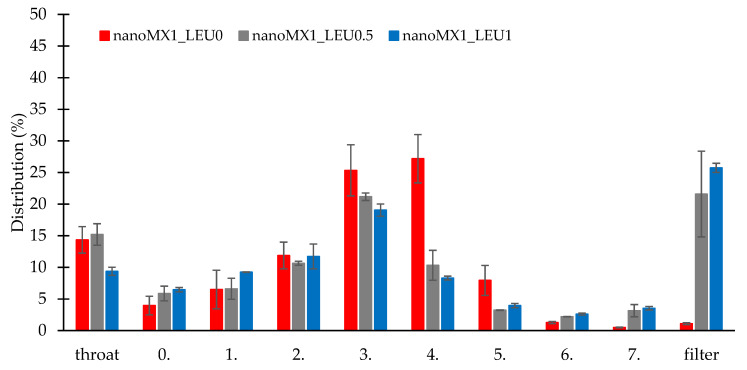
In vitro distribution of the samples (nanoMX1_LEU0, nanoMX1_LEU0.5, and nanoMX1_LEU1). Data are means ± SD (n = 3 independent measurements).

The in vitro aerodynamic results are demonstrated in Table 9. The MMAD values were between 1.55 and 2.33 µm, wherewith we could target the deeper airways [48]. The samples had FPF values between 72 and 76%, which is higher than the FPF values of the Breezhaler formulations on the market [32]. We can see the distribution of the products on the stages of the Andersen cascade impactor (Figure 6). The nanoMX1_LEU0 sample had 15% deposition in the upper airways, and 25% and 27% deposited on the third and fourth stages. The FPF value here was the most outstanding (75.67%). The application of LEU improved the aerosolization of the products owing to the reduced cohesion between the particles, which de-aggregated during inhalation. The samples had smaller MMAD (nanoMX1_LEU0.5: 1.74 µm, nanoMX1_LEU1: 1.55 µm) and were flown deeper in the ACI, deposited on the filter. We achieved high FPF values (nanoMX1_LEU0.5: 72.81%, nanoMX1_LEU0.5: 73.63%). The emitted fraction (EF) in most of the samples was also high (around 72–84%), indicating a weak adhesive character between the powder and the capsule, so a large amount of the product could be liberated from the device.

### 3.10. In Silico Aerodynamic Results

**Figure 7:**
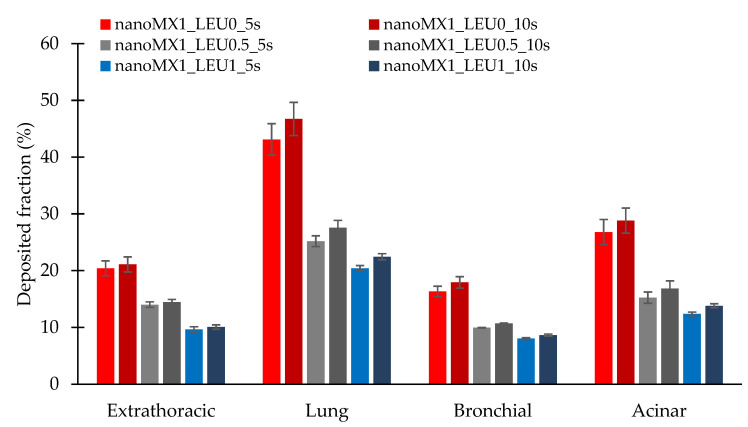
In silico aerodynamic results of the products (nanoMX1_LEU0, nanoMX1_LEU0_5s, nanoMX1_LEU0_10s, nanoMX1_LEU0.5_5s, nanoMX1_LEU0.5_10s, nanoMX1_LEU1_5s, and nanoMX1_LEU01_10s). Data are means ± SD (n = 3 independent measurements).

Figure 7 shows the deposition fractions of the samples within the extrathoracic airways and within different regions of the lung; that is, the bronchial and acinar pulmonary regions. The results were calculated with breath-holding time after inhalation of 5 s and 10 s. Using a breath-holding time of 10 s, the deposited fraction improved in all cases. The extrathoracic deposition is lower for the LEU containing products, thanks to improved dispersity. The lower deposition in the lung was because this method was defined as the deposited amount on the filter, as an exhaled fraction. The nanoMX1_LEU0 reached the highest deposition (47.47%) in the lung (Table 10). In all cases, higher values were obtained in the acinar region than in the bronchial region, which proved the delivery into the lower parts of the lung. It is a more proper approach to the real distribution in the airways than the in vitro method. However, these data are also promising for us because, in various lung diseases, the airways are usually damaged, contracted, or obstructed.

## 4. Discussion

The purpose of our research work was to develop a carrier-free “nano-in-micro” DPI system including the advantages of a nanonized active ingredient. We successfully worked out a “nano-in-micro” structured particle preparation method. We nanonized the API by wet milling and prepared micrometric sized particles by spray-drying. The samples containing MX, stabilizing additive (PVA), and aerosolization adjuvant (LEU) were characterized. From the nanosuspension, which contained MX nanoparticles (d = 137 nm), we managed to prepare nearly spherical microparticles with a size of 3–4 µm. By adding LEU, we could improve the yield (58%) of the spray-drying method. The specific surface area of the powders (1.7–2.2 m^2^/g) increased compared with the raw materials. With the low density (0.20–0.27 g/cm^3^) formulations, we achieved proper aerosolization properties. With the application of PVA, the polarity of the samples increased and, thanks to LEU, the cohesivity of the particles became lower. Part of the active ingredient was detected in an amorphous state according to the XRPD and DSC measurements. Owing to the particle size reduction, improved surface area, amorphization, and additives, dissolution was higher in the lung medium compared with the poorly water-soluble raw material, and the in vitro permeability of the samples also improved (61–87 µg/cm^2^/h). The dissolution and permeability results were beneficial to local delivery. Samples showed good aerosolization properties during the in vitro aerodynamic measurements: FPF above 72%, MMAD between 1.55 and 2.33 µm, and ED above 72%. The application of LEU increased the deposition in the deeper airways. The Andersen cascade impactor has limitations; that is, the data are usually higher than they could be in real circumstances [49]. The in silico aerodynamic values proved the deep deposition of the products in the respiratory airways. They showed higher deposition in the acinar region than in the bronchial region. This method is also just an approximated translation to patients.

## 5. Conclusions

The presented DPI offers an effective local treatment for lung diseases to prove it; it should be tested in vivo soon. The execution of the stability measurement is also important because of the “nano-in-micro” structure and the partial amorphization of the active ingredient.

## Figures and Tables

**Table 1:** Composition of the samples and the yield of spray-drying. MX, meloxicam; PVA, poly-vinyl-alcohol; LEU, l-leucine.

Samples	MX (g/L)	PVA (g/L)	LEU (g/L)	Yield * (%)
nanoMX1_LEU0	4.00	0.90	0.00	45.41 ± 5.10
nanoMX1_LEU0.5	4.00	0.90	2.00	57.56 ± 1.36
nanoMX1_LEU1	4.00	0.90	4.00	58.43 ± 6.36

* Data are means ± SD (n = 3 independent measurements).

**Table 2:** Composition of the physical mixtures.

Samples	MX (g)	PVA (g)	LEU (g)
pmMX1_LEU0	4.00	0.90	0.00
pmMX1_LEU0.5	4.00	0.90	2.00
pmMX1_LEU1	4.00	0.90	4.00

**Table 3:** Cut-off aerodynamic diameter for stages of Andersen cascade impactor (ACI) at a flow rate of 28.3 L/min.

ACI Stages	Cut-Off Diameter at 28.3 L/min (µm)
0	9.0–10.0
1	5.8–9.0
2	4.7–5.8
3	3.3–4.7
4	2.1–3.3
5	1.1–2.1
6	0.7–1.1
7	0.4–0.7
Filter	<0.4

**Table 4:** Particle size of the initial active pharmaceutical ingredient (API), the nanosuspension, the physical mixtures, and the final samples. SSA, specific surface area.

Samples	D[0.1] * (µm)	D[0.5] * (µm)	D[0.9] * (µm)	Span *	SSA * (m^2^/g)
raw MX	2.719 ± 0.057	9.913 ± 0.371	29.49 ± 0.630	2.70 ± 0.043	1.09 ± 0.028
MX suspension	0.067 ± 0.001	0.138 ± 0.005	0.555 ± 0.310	3.584 ± 2.056	43.65 ± 5.318
pmMX1_LEU0	3.073 ± 0.030	13.10 ± 0.500	349.92 ± 34.86	26.47 ± 1.649	0.88 ± 0.025
pmMX1_LEU0.5	5.426 ± 0.631	91.22 ± 17.90	357.57 ± 168.2	3.86 ± 1.101	0.40 ± 0.066
pmMX1_LEU1	7.983 ± 0.092	110.67 ± 0.261	353.25 ± 47.24	3.12 ± 0.433	0.27 ± 0.002
nanoMX1_LEU0	1.497 ± 0.046	3.186 ± 0.019	6.481 ± 0.193	1.56 ± 0.068	2.22 ± 0.031
nanoMX1_LEU0.5	1.834 ± 0.007	3.800 ± 0.014	7.389 ± 0.030	1.46 ± 0.004	1.88 ± 0.024
nanoMX1_LEU1	1.977 ± 0.093	4.396 ± 0.032	8.903 ± 0.186	1.58 ± 0.075	1.71 ± 0.051

* Data are means ± SD (n = 3 independent measurements).

**Table 5:** Diameter of the API in the products determined by Image-J analyses and the scanning electron microscopy (SEM) images of the spray-dried samples.

Samples	D * (nm)	SEM Pictures		
nanoMX1_LEU0	134.30 ± 23.07	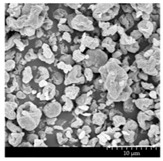	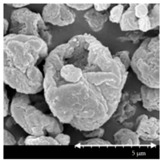	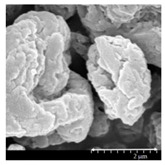
nanoMX1_LEU0.5	126.57 ± 27.26	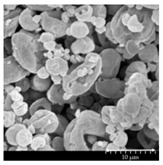	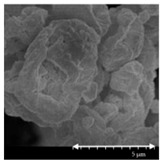	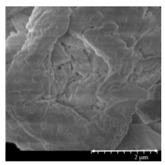
nanoMX1_LEU1	138.27 ± 42.57	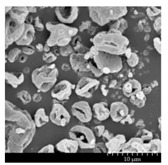	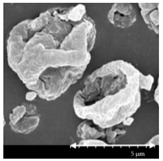	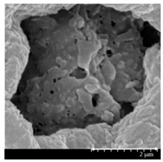

* Data are means ± SD (n = 100 independent measurements).

**Table 6:** Rheology properties of the samples.

Samples	Bulk Density * (g/cm^3^)	Tapped Density * (g/cm^3^)	Hausner Ratio *	Carr Index *	Flowability
nanoMX1_LEU0	0.177 ± 0.020	0.262 ± 0.001	1.488 ± 0.048	32.39 ± 7.232	Very poor
nanoMX1_LEU0.5	0.156 ± 0.009	0.274 ± 0.004	1.759 ± 0.084	43.09 ± 2.704	Very, very poor
nanoMX1_LEU1	0.147 ± 0.013	0.204 ± 0.012	1.398 ± 0.209	27.65 ± 10.82	Very poor

* Data are means ± SD (n = 3 independent measurements).

**Table 7:** Surface free energy, cohesion work, and polarity values of the samples and their components.

Samples	γ^d^ * [mN/m]	γ^p^ * [mN/m]	γ * [mN/m]	Wc * [mN/m]	Pol * [%]
MX	45.49 ± 0.09	13.89 ± 0.13	59.38 ± 0.22	118.76 ± 0.44	23.39 ± 0.15
PVA	45.65 ± 0.10	36.89 ± 0.20	82.54 ± 0.30	165.08 ± 0.60	44.69 ± 0.11
LEU	30.00 ± 0.07	0.50 ± 0.17	30.50 ± 0.24	61.00 ± 0.48	1.639 ± 0.20
pmMX1_LEU0	42.62 ± 0.12	30.65 ± 0.48	73.27 ± 0.60	146.54 ± 1.20	41.83 ± 0.56
pmMX1_LEU0.5	36.57 ± 0.34	25.63 ± 0.27	62.20 ± 0.61	124.40 ± 1.22	41.21 ± 0.84
pmMX1_LEU1	34.01 ± 0.55	16.57 ± 0.36	50.58 ± 0.91	101.16 ± 1.82	32.76 ± 0.44
nanoMX1_LEU0	42.34 ± 0.08	31.03 ± 0.62	73.38 ± 0.70	146.76 ± 1.40	42.29 ± 0.44
nanoMX1_LEU0.5	36.15 ± 0.95	25.69 ± 0.45	61.84 ± 0.51	123.68 ± 1.02	41.54 ± 1.07
nanoMX1_LEU1	33.39 ± 0.86	16.59 ± 0.11	49.98 ± 0.97	99.96 ± 1.94	33.19 ± 0.43

* Data are means ± SD (n = 3 independent measurements).

**Table 8:** In vitro permeability results of the samples.

Samples	J (µg/cm^2^/h)	Kp (cm/h)
rawMX	28.23	0.1394
pmMX1_LEU0	34.69	0.2081
pmMX1_LEU0.5	37.45	0.2247
pmMX1_LEU1	33.25	0.1995
nanoMX1_LEU0	61.80	0.3708
nanoMX1_LEU0.5	86.90	0.5214
nanoMX1_LEU1	73.58	0.4415

SD < ±2% (n = 3 independent measurements).

**Table 9:** In vitro aerodynamic properties of the “nano-in-micro” systems. FPD, fine particle dose; FPF, fine particle fraction; ED, emitted dose; EF, emitted fraction.

Samples	MMAD * (µm)	FPD * (mg)	FPF * (%)	ED * (mg)	EF * (%)	Loaded API * (mg)	API content * (%)
nanoMX1_LEU0	2.33 ± 0.08	4.52 ± 0.33	75.67 ± 3.46	5.98 ± 0.22	72.42 ± 3.05	8.26 ± 0.14	93.81 ± 2.99
nanoMX1_LEU0.5	1.74 ± 0.35	3.09 ± 0.31	72.81 ± 1.46	4.24 ± 0.34	83.47 ± 1.33	5.07 ± 0.33	55.48 ± 0.78
nanoMX1_LEU1	1.55 ± 0.06	2.51 ± 0.04	73.63 ± 0.96	3.40 ± 0.10	75.22 ± 1.75	4.53 ± 0.23	51.46 ± 0.66

* Data are means ± SD (n = 3 independent measurements).

**Table 10:** In silico aerodynamic properties with a breath-holding time of 10 s.

Samples	Deposited Fraction * (%)			
	Extrathoracic	Lung	Bronchial	Acinar
nanoMX1_LEU0	21.41 ± 2.79	46.73 ± 2.21	17.92 ± 2.93	28.81 ± 2.22
nanoMX1_LEU0.5	14.45 ± 0.95	27.55 ± 0.99	10.72 ± 1.30	16.83 ± 1.34
nanoMX1_LEU1	10.07 ± 0.47	22.44 ± 0.31	8.64 ± 0.54	13.80 ± 0.35

* Data are means ± SD (n = 3 independent measurements).
